# Long working hours are associated with unmet dental needs in south Korean male adults who have experienced dental pain

**DOI:** 10.1186/s12903-019-0953-8

**Published:** 2019-11-21

**Authors:** Yitak Kim, Sangwon Lee, Juyeong Kim, Eun-Cheol Park, Sung-In Jang

**Affiliations:** 1 College of Medicine, Yonsei University, Seoul, Republic of Korea; 2 Department of Health & Human Performance, Sahmyook University, Seoul, Republic of Korea; 3 Institute of Health Services Research, Yonsei University, Seoul, Republic of Korea; 4 Department of Preventive Medicine, Yonsei University College of Medicine, 50-1 Yonsei-ro, Seodaemun-gu, Seoul, 120-752 Republic of Korea

**Keywords:** Unmet needs, Dental pain, Unmet dental needs, Working hours, Dose-response relationship, Region, Alcohol

## Abstract

**Backgrounds:**

We explored the association between working hours and unmet dental needs among adults who have experienced dental pain, and how this relationship varied by demographic and lifestyle factors.

**Methods:**

We used the data of 9594 adults who reported dental pain from the Korea National Health and Nutrition Examination Survey (KNHANES) V and VI. We conducted a logistic regression analysis to determine the association between working hours and unmet dental needs, followed by a subgroup analysis and Cochran-Armitage trend tests.

**Results:**

Among the 4203 male subjects, 1661 (39.5%) experienced unmet dental needs. They also showed a significant dose-response relationship between working hours and unmet dental needs (OR 1.21 [95% CI 0.97–1.51], OR 1.30 [95% CI 0.99–1.69], OR 1.33 [95% CI 1.04–1.71], OR 1.58 [95% CI 1.21–2.07] compared to no working hours), whereas female participants did not. The significance of the association was preserved among participants with increased consumption of alcohol, urban residence, and who brushed their teeth at least twice a day. It was also stronger among those who lacked access to dental services or did not perceive the need for dental care.

**Conclusion:**

Among adults who have experienced dental pain, unmet dental needs had higher odds of occurring in males who worked longer, and this relationship appears to be influenced by consumption of alcohol, region of residence, tooth-brushing frequency, and access to and perception of dental care. Accordingly, policies should be drafted to reduce unmet needs by considering these factors.

## Background

A good society ensures that individuals can readily obtain appropriate medical services when needed. Accordingly, many countries strive to invest in medical facilities [1]. However, improving the medical facilities does not always translate to a better hospital experience for patients. In fact, quantitative expansion in medicine—such as constructing new hospitals and improving medical facilities—appears to have little effect on patients if patients cannot effectively reach a doctor [2].

A variety of obstacles can hinder individuals from reaching or deciding to contact a doctor, even when they might need to [3]. Unmet needs in health care can lead to a range of adverse health outcomes [4]. By identifying and resolving the causes of unmet needs in patients, we can expect an improvement in overall medical services without further investment in medical resources, which are often limited. Canada has noted a number of diverse efforts in considering gender, income, and social integration to alleviate inequalities in unmet dental needs [5].

In South Korea, the ratio of dental expenses to total medical expenses is rising rapidly, which accords with the dangerously high rate of unmet dental needs among both children and adults (20 and 40%, respectively) [6], Alarmingly, the rate of unmet dental needs is almost twice that of other diseases [6]. Thus, it is very important to determine and eliminate the factors that contribute to this high rate of unmet dental needs.

The excessive working hours among Korean adults is a contentious topic in Korean society. Among the Organization for Economic Co-operation and Development (OECD) countries, Korea was ranked third in terms of annual working hours in 2016 [7]. Many studies have pointed out that long working hours can have a range of adverse effects, including a higher incidence of physical problems such as diabetes mellitus and metabolic syndrome, as well as psychological problems such as anxiety and excessive alcohol use [8, 9]. It is similarly possible that longer working hours plays a role in unmet care needs, with overworking leading to less time available for accessing medical services. If this is found, reducing overall working hours might help improve the overall healthcare system.

There are several previous studies on the factors associated with unmet dental needs, conducted in both Western countries and South Korea [10, 11]. However, few of these studies utilized dental pain as an index of unmet dental needs, and most were limited to Western countries and child populations [10]. One study in the United States of America (USA) examined adults to find out factors of unmet dental needs, but they did not look for associations [12]. Also, studies examining the association between working hours and unmet dental needs among people who have experienced dental pain were generally unfound. This suggests the need for such study within this specific Korean population. By utilizing an objective index of illness (i.e., dental pain), we might be able to resolve the subjectivity of the unmet needs variable [13], which is often mentioned as a limitation in former studies [14, 15]. These objective indicators could certify the actual demands of patients, and more precisely detect health inequity. Therefore, we chose a sample exclusively comprised of subjects who have experienced dental pain, given that toothache is an unbiased index of the need for dental care [16].

We hypothesized that longer working hours will be associated with greater unmet needs among workers who have experienced dental pain. Furthermore, we performed a subgroup analysis with demographic and behavioral variables to examine which factors influence this association. Factors such as region of residence can affect the relationship, due to different accessibility to medical facilities.

## Methods

### Study design and participants

We used the data from the Korea National Health and Nutrition Examination Survey (KNHANES) V and VI, a nationwide cross-sectional study conducted from 2010 to 2015 by the Korean Ministry of Health and Welfare. The research population is homogeneous and unbiased, and represents non-institutionalized Korean civilians [17]. For this study, we selected adults older than 19 years old with valid responses to all items and had experienced dental pain. Among the 39,518 participants, after excluding participants with missing values and adolescents, we chose 10,118 respondents who reported the experience of dental pain. After further eliminating participants with invalid answers to regular dental checkups (*n* = 104), national health insurance (*n* = 85), drinking habits (*n* = 27), occupation (*n* = 305), and education (n = 3), 9594 respondents were analyzed.

### Working hours

Respondents were asked about their weekly working hours with the question “How long do you work per week, including extra work/night shift and excluding mealtimes?” The Labor Standards Act of Korea states that standard working hours must not exceed 40 h per week; work up to 48–60 h is defined as “extra work” and is given extra wages [18]. Studies have reported that people who work longer than 60 h per week tend to suffer from health problems such as higher cardiovascular mortality rates [19]. Accordingly, we classified participants’ answers to this question into four groups: 40 h or less, 41–48, 49–60, and 61 or more.

### Unmet needs

Unmet dental needs were assessed with two questions. First, they were asked “Have you ever wanted to visit a dentist but could not?”. Those who answered ‘yes’ were then asked why, and the answers were classified into three groups. Firstly, ‘Lack of ability to pay’ was classified as ‘economic reasons’. Secondly, ‘Dental clinic is too far away,’ ‘could not leave workplace or school,’ ‘mobility or health problems,’ and ‘had to take care of children’ were classified as ‘lack of access’. Finally, ‘did not consider it a serious problem’ and ‘afraid to visit a dentist’ were classified as ‘perceptual barriers’ [20].

### Other variables

Demographic variables included gender, age (20–29, 30–39, 40–49, 50–59, 60–65, and > 65 years), and region of residence (urban or rural areas). The socioeconomic variables included level of education (middle school or lower, high school, and college or higher), occupation type (office, labor, and service), and household income (high, moderately high, moderately low, and low). Household income was divided into quartiles using the monthly average equivalent household income (i.e., monthly household income divided by the square root of the number of household members) [17]. The health-related variables considered were smoking (non-smoker, past smoker, and present smoker), drinking habits (non-drinker, light drinker, and heavy drinker), possession of private insurance, health insurance type (national health insurance (NHI)-local, NHI-employee’s, and medical aid), self-rated oral health status (good, moderate, bad). Heavy drinkers were defined as those who drank > 2 times a week, light drinkers as those who drank less than twice per week, and non-drinkers as those who never drank or drank less than once per month. Finally, the dental care indicators included usage of dental care tools, number of times of teeth brushing per day, and dental checkup within the last year. Unless otherwise mentioned, the above variables were binary variables (yes or no).

### Statistical analysis

Because average workload, occupation, and physical abilities differ considerably according to gender [21], we stratified all analysis by gender. For binary variables, we calculated the frequency and proportions of each variable and compared them using chi-square tests. The association was quantified using logistic regression analyses after adjusting for demographic, socioeconomic, health-related, and dental care indicators. Additionally, we performed subgroup analyses according to drinking habits, region of residence, and tooth-brushing habit. Cochran–Armitage trend tests were used to determine the p for trend between working hours and unmet dental needs. For this test, working hours were defined as a continuous variable (with an interval of 1 h) and unmet dental needs as a binary variable. All analyses were conducted using SAS 9.4 (SAS Inc., Cary, NC, USA). There were no human subjects involved in this study.

## Results

Table 1 displays the general characteristics of the gender-stratified study population. Of the 4203 (43.8%) male participants and 5391 (56.2%) female participants, 1661 (39.5%) and 2376 (44.1%) had experienced unmet dental needs, respectively. Among both males and females, the percentage of unmet dental needs increased with working hours (*p* < 0.001 in males, *p* = 0.017 in female). Specifically, among males, the proportions of unmet dental needs were 32.3, 38.5, 40.9, 41.7, and 46.9% in the 0, < 40, 41–48, 49–60, and > 60 h groups, respectively; among females, the proportions of unmet dental needs were 41.3, 45.2, 46.2, 46.3, and 48.2%, respectively.

**Table 1:** General characteristics by unmet dental need

Variable	N	%	Male (*n* = 4173)					Female (*n* = 5355)				
			No unmet need		Unmet need			No unmet need		Unmet need		
			N	%	N	%	*p*-value	N	%	N	%	*p-*value
Total	9528	100.0	2524	60.5	1649	39.5		2995	55.9	2360	44.1	
Working hours per week							< 0.001					0.030
No work	2816	29.6	510	67.5	245	32.5		1208	58.6	853	41.4	
< 40	3346	35.1	844	61.6	527	38.4		1083	54.8	892	45.2	
41–48	1100	11.5	365	59.1	253	40.9		259	53.7	223	46.3	
49–60	1366	14.3	516	58.2	371	41.8		258	53.9	221	46.1	
> 60	900	9.4	289	53.3	253	46.7		187	52.2	171	47.8	
Age							< 0.001					0.160
20–29	1076	11.3	245	59.3	168	40.7		353	53.2	310	46.8	
30–39	1678	17.6	370	54.2	313	45.8		537	54.0	458	46.0	
40–49	1692	17.8	448	59.7	303	40.3		539	57.3	402	42.7	
50–59	2019	21.2	527	56.4	407	43.6		595	54.8	490	45.2	
60–65	1058	11.1	311	64.8	169	35.2		335	58.0	243	42.0	
65-	2005	21.0	623	68.3	289	31.7		636	58.2	457	41.8	
Private insurance							0.404					0.184
No	2551	26.8	711	61.5	445	38.5		759	54.4	636	45.6	
Yes	6977	73.2	1813	60.1	1204	39.9		2236	56.5	1724	43.5	
Health insurance type							< 0.001					< 0.001
NHI (local)	3215	33.7	841	57.4	623	42.6		921	52.6	830	47.4	
NHI (employee’s)	5971	62.7	1629	62.7	969	37.3		1966	58.3	1407	41.7	
Medical aid	342	3.6	54	48.6	57	51.4		108	46.8	123	53.2	
Household income							0.035					< 0.001
Low	1817	19.1	418	58.0	303	42.0		564	51.5	532	48.5	
low - moderate	2471	25.9	634	59.9	425	40.1		764	54.1	648	45.9	
Moderate -High	2599	27.3	693	59.1	479	40.9		816	57.2	611	42.8	
High	2641	27.7	779	63.8	442	36.2		851	59.9	569	40.1	
Region of residence							0.001					0.736
Urban	7417	77.8	2005	61.8	1239	38.2		2339	56.1	1834	43.9	
Rural	2111	22.2	519	55.9	410	44.1		656	55.5	526	44.5	
Occupation type							0.065					0.308
Office	3417	35.9	919	62.8	544	37.2		1108	56.7	846	43.3	
Labor	5181	54.4	1464	59.1	1015	40.9		1514	56.0	1188	44.0	
Service	930	9.8	141	61.0	90	39.0		373	53.4	326	46.6	
Self-assessment of dental health							< 0.001					< 0.001
Good	716	7.5	297	83.4	59	16.6		286	79.4	74	20.6	
Moderate	3071	32.2	890	71.9	347	28.1		1226	66.8	608	33.2	
Bad	5741	60.3	1337	51.8	1243	48.2		1483	46.9	1678	53.1	
Reason for unmet dental needs*							< 0.001					< 0.001
No unmet need	5519	57.9	2524	100.0	0	0.0		2995	100.0	0	0.0	
Lack of ability to pay			0	0.0	590	35.8		0	0.0	931	39.4	
Lack of ability to reach			0	0.0	543	32.9		0	0.0	617	43.2	
Lack of ability to perceive			0	0.0	516	31.3		0	0.0	812	28.1	
Number of family members							0.018					0.063
More than one	8654	90.8	2367	61.0	1515	39.0		2690	56.4	2082	43.6	
Alone	874	9.2	157	54.0	134	46.0		305	52.3	278	47.7	
Level of education							0.020					0.644
Middle school	3457	36.3	758	60.8	488	39.2		1223	55.3	988	44.7	
High school	3049	32.0	829	57.8	606	42.2		902	55.9	712	44.1	
≥ college	3022	31.7	937	62.8	555	37.2		870	56.9	660	43.1	
Usage of dental care tools							< 0.001					< 0.001
No	5363	56.3	1481	56.9	1120	43.1		1423	51.5	1339	48.5	
Yes	4165	43.7	1043	66.3	529	33.7		1572	60.6	1021	39.4	
Number of times brushing teeth per day			0.004					0.069				
0–1	1240	13.0	419	55.9	331	44.1		255	52.0	235	48.0	
≥ 2	8288	87.0	2105	61.5	1318	38.5		2740	56.3	2125	43.7	
Dental checkup within last one year							< 0.001					< 0.001
No	6546	68.7	1596	56.9	1207	43.1		1900	50.8	1843	49.2	
Yes	2982	31.3	928	67.7	442	32.3		1095	67.9	517	32.1	
Smoke							< 0.001					< 0.001
No	5424	56.9	500	67.1	245	32.9		2666	57.0	2013	43.0	
Current smoker	2104	22.1	947	53.7	815	46.3		162	47.4	180	52.6	
Past smoker	2000	21.0	1077	64.6	589	35.4		167	50.0	167	50.0	
Drink							0.148					0.938
No drink	3579	37.6	444	61.5	278	38.5		1110	56.2	864	43.8	
Light drink	2363	24.8	1135	61.7	704	38.3		1594	55.8	1263	44.2	
Heavy drink	2136	22.4	945	58.6	667	41.4		291	55.5	233	44.5	
Average hours of sleep per week							0.036					< 0.001
< 5	478	5.0	86	55.1	70	44.9		159	49.4	163	50.6	
5–6	3772	39.6	980	58.4	698	41.6		1125	53.7	969	46.3	
7–8	4588	48.2	1297	62.5	777	37.5		1474	58.6	1040	41.4	
> 8	690	7.2	161	60.8	104	39.2		237	55.8	188	44.2	
BMI							0.113					0.130
≤ 25	4275	44.9	1552	59.6	1054	40.4		2087	56.6	1599	43.4	
> 25	3236	34.0	972	62.0	595	38.0		908	54.4	761	45.6	
Year							< 0.001					< 0.001
2010	1489	15.6	357	54.0	304	46.0		419	50.6	409	49.4	
2011	1335	14.0	329	57.1	247	42.9		408	53.8	351	46.2	
2012	1789	18.8	459	58.2	329	41.8		530	52.9	471	47.1	
2013	1769	18.6	486	62.6	290	37.4		589	59.3	404	40.7	
2014	1529	16.0	448	67.4	217	32.6		497	57.5	367	42.5	
2015	1617	17.0	445	62.9	262	37.1		552	60.7	358	39.3	

* The ratios of each reason represents the percentage compared to the number of people who showed unmet needs

Table 2 presents the logistic regression analysis results adjusted with confounding factors for both males and females. We observed a dose-response relationship between working hours and unmet need *only* in male participants. Specifically, the odds ratios (ORs) and 95% confidence intervals (CI) for the working hour groups (vs. the 0-h group) were as follows: OR = 1.21 [95% CI 0.97–1.51] for < 40 h; OR = 1.30 [95% CI 0.99–1.69] for 41–48 h; OR = 1.33 [95% CI 1.04–1.71] for 49–60 h; and OR = 1.58 [95% CI 1.21–2.07] for > 60 h. In other words, the odds ratios increased with working hours among males. Among females, the ORs showed a bell-shaped pattern (OR = 1.24 [95% CI 1.08–1.42] for < 40 h; OR = 1.27 [95% CI 1.02–1.58] for 41–48 h; OR = 1.24 [95% CI 1.00–1.54] for 49–60 h; OR = 1.21 [95% CI 0.95–1.55] for > 60 h).

**Table 2:** Adjusted odds ratios for factors associated with unmet dental need

Variable	Male (p < 0.001)*			Female (*p* = 0.001)*		
	Unmet need			Unmet need		
	OR	95% CI		OR	95% CI	
Working hours per week						
no work	1.00			1.00		
< 40	1.21	0.97	1.51	1.23	1.07	1.41
41–48	1.29	0.98	1.68	1.26	1.01	1.56
49–60	1.32	1.03	1.71	1.22	0.98	1.52
> 60	1.54	1.17	2.02	1.16	0.90	1.48
Age						
20–29	1.00			1.00		
30–39	1.24	0.94	1.64	1.04	0.84	1.29
40–49	0.92	0.70	1.21	0.90	0.72	1.12
50–59	1.00	0.76	1.31	0.99	0.78	1.26
60–65	0.73	0.53	1.00	0.78	0.58	1.04
65-	0.57	0.42	0.78	0.63	0.47	0.85
Private insurance						
No	1.00			1.00		
Yes	0.97	0.81	1.17	1.00	0.85	1.17
Health insurance type						
NHI (local)	1.00			1.00		
NHI (employee’s)	0.90	0.78	1.04	0.86	0.76	0.97
Medical aid	1.33	0.86	2.06	1.16	0.85	1.57
Household income						
Low	1.00			1.00		
low - moderate	0.85	0.68	1.07	0.89	0.74	1.08
Moderate -High	0.89	0.70	1.12	0.81	0.66	0.99
High	0.76	0.59	0.97	0.79	0.64	0.97
Region of residence						
Urban	1.00			1.00		
Rural	1.26	1.07	1.48	0.95	0.82	1.09
Occupation type						
Office	1.00			1.00		
Labor	0.93	0.78	1.10	0.84	0.70	1.00
Service	0.84	0.61	1.16	0.95	0.77	1.17
Self-assessment of dental health						
Good	1.00			1.00		
Moderate	1.80	1.31	2.46	1.94	1.47	2.56
Bad	4.22	3.14	5.68	4.41	3.36	5.78
Number of family members						
More than one	1.00			1.00		
Alone	1.21	0.93	1.58	1.11	0.91	1.36
Level of education						
Middle school	1.00			1.00		
High school	1.18	0.97	1.43	1.08	0.89	1.31
≥ college	1.05	0.84	1.32	1.16	0.91	1.48
Usage of dental care tools						
No	1.00			1.00		
Yes	0.74	0.64	0.86	0.75	0.66	0.85
Number of times brushing teeth per day						
0–1	1.00			1.00		
≥ 2	0.85	0.72	1.02	0.89	0.73	1.10
Dental checkup within last one year						
No	1.00			1.00		
Yes	0.68	0.59	0.79	0.51	0.44	0.58
Smoke						
No	1.00			1.00		
Current smoker	1.39	1.15	1.70	1.20	0.94	1.53
Past smoker	1.19	0.97	1.45	1.28	1.01	1.62
Drink						
No drink	1.00			1.00		
Light drink	0.87	0.71	1.05	0.96	0.84	1.09
Heavy drink	0.89	0.73	1.09	0.87	0.70	1.07
Average hours of sleep per week						
< 5	1.00			1.00		
5–6	0.80	0.56	1.14	0.88	0.68	1.13
7–8	0.68	0.48	0.97	0.72	0.56	0.93
> 8	0.67	0.44	1.03	0.72	0.52	0.98
BMI						
≤ 25	1.00			1.00		
> 25	0.89	0.78	1.02	1.03	0.91	1.17
Year						
2010	1.21	0.96	1.53	1.28	1.05	1.57
2011	1.11	0.87	1.41	1.17	0.95	1.44
2012	1.10	0.88	1.38	1.29	1.06	1.56
2013	0.94	0.75	1.18	1.06	0.87	1.28
2014	0.81	0.64	1.02	1.15	0.94	1.40
2015	1.00			1.00		

* These *p*-values represent the result of the Cochran-Armitage trend test for each subgroup

The subgroup analysis is shown in Table 3, separately for males and females. For alcohol consumption, males defined as heavy drinkers showed significantly higher ORs (for the 41–48, 49–60, and > 60 h) and maintained the dose-response relationship. Among females, the ORs increased with working hours in the light drinking group particularly, although significance was found only for the 1–40, 41–48, and 49–60 h groups. As for region of residence, males continued to show a dose-response relationship in urban areas (OR = 1.17 [95% CI 0.90–1.51] for < 40 h; OR = 1.39 [95% CI 1.02–1.90] for 41–48 h; OR = 1.52 [95% CI 1.14–2.03] for 49–60 h; OR = 1.65 [95% CI 1.21–2.25] for > 60 h), but not in rural areas. As for times tooth-brushing habit, both males and females showed a stronger positive relationship between working hours and unmet dental need when they brushed at least twice a day.

**Table 3:** Association between work hours and unmet need by different factors

Variable		Male			Female		
		Unmet need			Unmet need		
		OR	95% CI		OR	95% CI	
Drink							
No drink	0 h	1.00	***p*** = 0.011*			***p*** **= 0.066**	
	1–40 h	0.99	0.62	1.58	1.19	0.95	1.48
	40–48 h	1.32	0.70	2.50	1.06	0.72	1.58
	48–60 h	1.10	0.61	1.98	1.00	0.66	1.51
	≥61 h	1.71	0.91	3.24	1.42	0.93	2.19
Light drink	0 h	1.00	***p*** **= 0.002**			***p*** **= 0.007**	
	1–40 h	1.15	0.82	1.62	1.27	1.05	1.54
	40–48 h	1.12	0.75	1.68	1.38	1.03	1.85
	48–60 h	1.23	0.84	1.80	1.44	1.07	1.93
	≥61 h	1.56	1.02	2.39	1.11	0.78	1.56
Heavy drink	0 h	1.00	**p < 0.001**			***p*** **= 0.204**	
	1–40 h	1.44	0.97	2.15	1.19	0.74	1.91
	40–48 h	1.61	1.01	2.57	1.43	0.73	2.81
	48–60 h	1.69	1.08	2.65	1.07	0.55	2.08
	≥61 h	1.63	1.03	2.59	0.84	0.40	1.76
Region of residence							
Urban	0 h	1.00	**p < 0.001**			**p = 0.002**	
	1–40 h	1.16	0.90	1.51	1.20	1.02	1.40
	40–48 h	1.37	1.01	1.87	1.22	0.95	1.55
	48–60 h	1.53	1.14	2.04	1.31	1.01	1.70
	≥61 h	1.60	1.17	2.18	1.12	0.83	1.51
Rural	0 h	1.00	***p*** **= 0.114**			***p*** **= 0.215**	
	1–40 h	1.35	0.86	2.13	1.47	1.08	1.98
	40–48 h	1.00	0.57	1.74	1.54	0.95	2.50
	48–60 h	0.83	0.49	1.42	1.28	0.83	1.96
	≥61 h	1.39	0.79	2.45	1.44	0.90	2.29
Number of times brushing teeth per day							
0–1	0 h	1.00	***p*** **= 0.019**			***p*** **= 0.362**	
	1–40 h	1.41	0.89	2.23	1.01	0.64	1.59
	40–48 h	1.17	0.62	2.20	0.85	0.33	2.18
	48–60 h	1.20	0.67	2.16	0.78	0.36	1.68
	≥61 h	1.67	0.93	3.00	1.37	0.65	2.89
≥ 2	0 h	1.00	**p < 0.001**			**p = 0.001**	
	1–40 h	1.18	0.91	1.52	1.26	1.09	1.46
	40–48 h	1.30	0.96	1.76	1.30	1.04	1.63
	48–60 h	1.37	1.03	1.83	1.30	1.03	1.63
	≥61 h	1.56	1.14	2.13	1.15	0.88	1.50

* Bolded numbers represent the p-value of the Cochran-Armitage trend test for each subgroup

Table 4 shows the associations between working hours and unmet dental needs for each reason group. For both males and females, participants who lacked access to dental care and had perceptual barriers for dental care showed higher ORs than did those who lacked the ability to pay. Furthermore, among participants who lacked access, the relationship between working hours and unmet dental needs remained positive; in contrast, the relationship was negative among those who had perceptual barriers for dental care.

**Table 4:** Association by different reasons of unmet need

Variable		Male			Female		
		Unmet need			Unmet need		
		OR	95% CI		OR	95% CI	
Reasons of unmet need							
Lack of ability to pay	0 h	1.00	< 0.001*		1.00	**< 0.001**	
	1–40 h	1.08	0.74	1.58	1.08	0.86	1.35
	40–48 h	1.26	0.80	1.98	1.08	0.76	1.54
	48–60 h	0.89	0.57	1.37	0.69	0.48	0.99
	≥61 h	1.26	0.81	1.97	0.84	0.58	1.23
Lack of ability to reach	0 h	1.00	**< 0.001**		1.00	**< 0.001**	
	1–40 h	1.43	0.91	2.24	1.14	0.89	1.46
	40–48 h	1.68	1.02	2.76	2.12	1.50	2.98
	48–60 h	2.80	1.75	4.48	3.21	2.28	4.51
	≥61 h	2.36	1.45	3.84	2.30	1.56	3.40
Lack of ability to perceive	0 h	1.00	**0.004**		1.00	**< 0.001**	
	1–40 h	0.70	0.48	1.03	0.81	0.65	1.01
	40–48 h	0.53	0.34	0.83	0.45	0.32	0.64
	48–60 h	0.40	0.26	0.62	0.43	0.30	0.61
	≥61 h	0.35	0.22	0.56	0.54	0.36	0.80

* Bolded numbers represent the p-value of the Cochran-Armitage trend test for each subgroup

## Discussion

The purpose of this study was to examine the association between working hours and unmet dental needs in a specific population of Koreans—those with experience of dental pain. We also conducted subgroup analyses by alcohol consumption level, region of residence, tooth-brushing frequency, and the major reasons for unmet dental needs.

We observed a dose-response relationship between working hours and unmet dental needs in the male group. In order to interpret effect sizes of odds ratios, the odds ratios were converted into Cohen’s d, or the standardized mean difference between two group means [22]. While the effect size of the odds ratio of the > 60 h group (OR = 1.54, or Cohen’s d = 0.2) is considered small, it is nevertheless reflective of a continued stepwise association among males. No association between working hours and unmet dental needs was observed for females. The potential cause of this relationship in males is the early closing time of hospitals in South Korea. In other words, when a salaried worker visits the hospital after work (usually at 5 pm), there is little time for them to see a doctor before the hospital closes (typically around 6 pm). Given that South Korea has long working hours (ranked 3rd place among OECD countries) [7], it is feasible that longer working hours leads to greater unmet needs.

Unlike a previous study [15], females did not show a positive relationship between working hours and unmet care needs. However, participants in this study all had experienced dental pain, unlike of participants in Soek et al. The time periods when the data was collected are also different. The differing results suggest the validity of gender stratification in studies on employment status [21, 23]. Gender difference in workplaces can also lead to different job roles and positions, even when the occupation type and working hours are the same [21]. Greater unmet needs can imply either of the following: higher unmet with identical needs, or superior demands in the first place. The former might be explained by conventional gender roles, whereby men tend to gain more stress from loss of job opportunities and job-related failures [24]. Accordingly, men might experience greater pressure at their workplaces, which hinders them from taking sick leave. The latter can be understood by considering that males have higher health-related concerns, including metabolic syndromes and suicide rates [23, 25]. Regardless of the explanations, stricter regulation policies for working hours appear to be more necessary for males.

The subgroup analysis for alcohol consumption in Table 3 indicates that there are generally significant associations between working hours and unmet dental needs among heavy drinkers for males and light drinkers for females. Drinking habits are linked to decreased risk awareness, and alcohol-related disorders require social treatments [26, 27]. Thus, excessive alcohol consumption can mislead people from receiving proper medication, which relates to the significance of the results. The higher ORs in the light drinking group among females is perhaps due to the lower tolerance of alcohol in women compared to men [28].

Region of residence has been highlighted as a controversial factor in recent years [29–31]. Rural areas have markedly different characteristics from urban areas, including a more restricted labor market, closed social network, and limited health-related resources [30]. Previous studies have shown both higher and comparable levels of unmet needs in rural regions (vs. urban ones) among Americans [30, 31]. Conversely, a study in the Korean population claimed that urban residents experience greater unmet care needs for outpatient care, after adjusting for other factors [29]. These discrepancies potentially result from the differing cultures and healthcare systems. Our findings are consistent with Kim et al.’s [29], with the urban subgroup showing a significant dose-response relationship. Despite the proximity of hospitals in urban regions, urban residents’ lack of time might lead to greater unmet needs [15].

Interestingly, participants with a higher frequency of tooth brushing showed greater ORs and stronger relationship between working hours and unmet dental needs. This contradicts the commonsense notion that regular tooth brushing improves oral health [32]. However, in this study, more brushing might imply greater effort to improve health status, since all participants had experienced dental pain. These individuals might have higher expectations in their health, thus elevating their demand and strengthening the relationship between working hours and dental unmet need. Past studies support this idea, where privately insured people showed greater unmet needs [33].

In this study, lack of access and perceptual barriers for dental care were significant moderators of the relationship between working hours and unmet dental need. These results are consistent with previous papers demonstrating that a lack of time is a major reason for unmet needs [15, 33]. The negative relationship between working hours and unmet needs in those who had perceptual barriers for dental care can be explained by the subjectivity of unmet needs, which are highly personal [34]. Specifically, when an unmet need is not perceived as a need by the person—for instance, when individuals perceive working is more important than visiting the doctor—the actual demand will decrease, thus leading to the negative association between working hours and unmet needs. The fact that unmet needs are not solely the result of economic burden supports the idea that society itself must do more to improve overall healthcare quality than mere financial investment.

Our results have several policy implications. First, the association between working hours and unmet dental needs, and the fact that lack of access (including time) is a major reason for unmet, indicate the importance of time in health care. To ensure adequate health care access, policies should be put in place to reduce time-related barriers, such as extending sick-leave breaks or changing hospital hours. Region should also be considered in these policies. Previous studies in Canada and South Ethiopia evaluating differences along the rural–urban continuum have sought to utilize primary health care systems to reduce unmet needs [35, 36]. These former findings suggest feasible strategies for urban areas with long working hours. Lastly, this study is also likely to support the field of preventive dentistry by precluding unmet dental needs in advance.

This study has several limitations. First, cross-sectional data cannot infer any causal relationships. Therefore, caution is required in interpreting our results. Second, this study excluded relevant occupation-related variables: the sample was stratified according to working hours (including non-working group) but did not account for nightshift work or wage type due to collinearity. Further research should include these variables as confounders. Third, we did not include all possible oral health-related factors, such as history of dental diseases, oral health education and medication, because these specific variables were not included in KNHANES questionnaire. Lastly, our manipulation of the occupation variable might disturb the validity of the results; while it preserved 30% of the population, further studies are needed to prove its validity.

Nevertheless, this study endeavored to remain nationally representative by utilizing 6 years of longitudinal data and a large sample. In addition, the sample contained non-workers and all had experience of dental pain, meaning that the population was refined. Furthermore, plausible mechanisms for obscure issues were demonstrated by taking advantage of up-to-date evidence.

## Conclusion

Only male subjects showed significant dose-response relationships between working hours and unmet dental needs. In addition, increased consumption of alcohol, residing in urban areas, brushing teeth at least twice a day, and lacking access and perception of the need for dental care significantly moderated the relationships between working hours and unmet dental needs. Future research controlling for other occupation variables would further solidify our results and offer more practical implications for workplaces in South Korea.

## Data Availability

KNHANES V and VI data can be accessed from the KNHANES homepage (URL: https://knhanes.cdc.go.kr/knhanes/eng/index.do).

## Abbreviations

CI: Confidence Interval
h, hr.: hour
KNHANES: Korea National Health and Nutrition Examination Survey
NHI: National Health Insurance
OECD: Organization for Economic Co-Operation and Development
OR: Odds Ratio
USA: United States of America

## References

[1] Kapferer S (2015). **The importance of investing in health**. In.

[2] Staff Writer (2014). TOP 10: countries investing the Most on health care.

[3] Sanmartin C, Houle C, Tremblay S, Berthelot JM (2002). Changes in unmet health care needs. Health Rep.

[4] Ko H (2016). Unmet healthcare needs and health status: panel evidence from Korea. Health Policy.

[5] Bryant T, Leaver C, Dunn J (2009). Unmet healthcare need, gender, and health inequalities in Canada. Health Policy.

[6] Jung H: Korean National Health Accounts in 2014. In*.* Edited by Ministry of Health and Welfare: Ministry of Health and Welfare,; 2015.

[7] Hours worked. https://data.oecd.org/emp/hours-worked.htm.

[8] Kivimaki M, Virtanen M, Kawachi I, Nyberg ST, Alfredsson L, Batty GD, Bjorner JB, Borritz M, Brunner EJ, Burr H (2015). Long working hours, socioeconomic status, and the risk of incident type 2 diabetes: a meta-analysis of published and unpublished data from 222 120 individuals. The lancet Diabetes & endocrinology.

[9] Bannai A, Tamakoshi A (2014). The association between long working hours and health: a systematic review of epidemiological evidence. Scand J Work Environ Health.

[10] Villalobos-Rodelo JJ, Medina-Solis CE, Maupome G, Lamadrid-Figueroa H, Casanova-Rosado AJ, Casanova-Rosado JF, Marquez-Corona Mde L (2010). Dental needs and socioeconomic status associated with utilization of dental services in the presence of dental pain: a case-control study in children. J Orofac Pain.

[11] Chae S (2017). Factors associated with perceived unmet dental care needs of older adults unmet dental care needs. Geriatr Gerontol Int.

[12] Caban-Martinez AJ, Lee DJ, Fleming LE, Arheart KL, LeBlanc WG, Chung-Bridges K, Christ S, Pitman T: Dental care access and unmet dental care needs among U.S. workers: the National Health Interview Survey, 1997 to 2003. The Journal of the American Dental Association 2007, 138(2):227–230. 10.14219/jada.archive.2007.0141 17272379

[13] Bebbington PE, Meltzer H, Brugha TS, Farrell M, Jenkins R, Ceresa C, Lewis G (2000). Unequal access and unmet need: neurotic disorders and the use of primary care services. Psychol Med.

[14] Popovic N, Terzic-Supic Z, Simic S, Mladenovic B (2017). Predictors of unmet health care needs in Serbia; analysis based on EU-SILC data. PLoS One.

[15] Soek H (2016). A dose–response relationship between long working hours and unmet need for access to hospital facilities. Scand J Work Environ Health.

[16] Lewis C, Stout J (2010). Toothache in us children. Archives of Pediatrics & Adolescent Medicine.

[17] Kweon S, Kim Y (2014). Jang M-j, Kim Y, Kim K, Choi S, Chun C, Khang Y-H, oh K: **data resource profile: the Korea National Health and nutrition examination survey (KNHANES)**. Int J Epidemiol.

[18] Ministry of Government Legislation: Labor Standards Act. In*.* Edited by Ministry of Employment and Labor. Seoul; 2010: Article 50.

[19] Spurgeon A, Harrington JM, Cooper CL (1997). Health and safety problems associated with long working hours: a review of the current position. Occup Environ Med.

[20] Levesque JF, Harris MF, Russell G (2013). Patient-centred access to health care: conceptualising access at the interface of health systems and populations. Int J Equity Health.

[21] Park JW, Park JS, Kim S, Park M, Choi H, Lim S (2016). The association between long working hours and hearing impairment in noise unexposed workers: data from the 5th Korea National Health and nutrition examination survey (KNHANES 2010-2012). Annals of occupational and environmental medicine.

[22] Chen H, Cohen P, Chen S (2010). How big is a big odds ratio? Interpreting the magnitudes of odds ratios in epidemiological studies. Communications in Statistics - Simulation and Computation.

[23] Myong J-P, Kim H-R, Jung-Choi K, Baker D, Choi B (2012). Disparities of metabolic syndrome prevalence by age, gender and occupation among Korean adult workers. Ind Health.

[24] Cutright P (2001). The relative gender gap in suicide: societal integration, the culture of suicide, and period effects in 20 developed countries, 1955–1994. Soc Sci Res.

[25] Kim SY (2011). Comparative epidemiology of suicide in South Korea and Japan: effects of age, gender and suicide methods. Crisis : the journal of crisis intervention and suicide prevention.

[26] Steptoe A, Wardle J (2001). Health behaviour, risk awareness and emotional well-being in students from Eastern Europe and Western Europe. Soc Sci Med.

[27] Mojtabai R, Crum RM (2013). Perceived unmet need for alcohol and drug use treatments and future use of services: results from a longitudinal study. Drug Alcohol Depend.

[28] Nolen-Hoeksema S (2004). Gender differences in risk factors and consequences for alcohol use and problems. Clin Psychol Rev.

[29] Kim J, Kim TH, Park EC, Cho WH: Factors influencing unmet need for health care services in Korea. *Asia-Pacific journal of public health* 2015, **27**(2):Np2555–2569. 10.1177/1010539513490789 23858512

[30] Peterson: County-Level Poverty Is Equally Associated With Unmet Health Care Needs in Rural and Urban Settings. *The Journal of rural health* 2010, **26**(4):373. 10.1111/j.1748-0361.2010.00309.x 21029173

[31] Merwin E, Snyder A, Katz E (2006). Differential access to quality rural healthcare: professional and policy challenges. Family & Community Health.

[32] Buglar ME, White KM, Robinson NG (2010). The role of self-efficacy in dental patients’ brushing and flossing: testing an extended health belief model. Patient Educ Couns.

[33] Lee S-Y (2015). Unmet healthcare needs depending on employment status. Health policy (Amsterdam).

[34] Allin S: Subjective unmet need and utilization of health care services in Canada: What are the equity implications? *Social science & medicine (1982)* 2010, **70**(3):465–472. 10.1016/j.socscimed.2009.10.027 19914759

[35] Sibley LM, Weiner JP (2011). An evaluation of access to health care services along the rural-urban continuum in Canada. BMC Health Serv Res.

[36] Mekonnen W, Worku A: Determinants of low family planning use and high unmet need in Butajira District**,** South Central Ethiopia *Reproductive Health* 2011, 8(1):37. 10.1186/1742-4755-8-37 PMC3248357 22151888

